# Inhibitory synaptic loss drives network changes in multiple sclerosis: An ex vivo to in silico translational study

**DOI:** 10.1177/13524585221125381

**Published:** 2022-10-03

**Authors:** Marijn Huiskamp, Svenja Kiljan, Shanna Kulik, Maarteen E Witte, Laura E Jonkman, John GJM Bol, Geert J Schenk, Hanneke E Hulst, Prejaas Tewarie, Menno M Schoonheim, Jeroen JG Geurts

**Affiliations:** Anatomy and Neurosciences, MS Center Amsterdam, Vrije Universiteit Amsterdam, Amsterdam Neuroscience, Amsterdam UMC location VUmc, De Boelelaan 1108, 1081 HZ Amsterdam, The Netherlands; Anatomy and Neurosciences, MS Center Amsterdam, Vrije Universiteit Amsterdam, Amsterdam Neuroscience, Amsterdam UMC location VUmc, Amsterdam, The Netherlands; Anatomy and Neurosciences, MS Center Amsterdam, Vrije Universiteit Amsterdam, Amsterdam Neuroscience, Amsterdam UMC location VUmc, Amsterdam, The Netherlands; Molecular Cell Biology and Inflammation, MS Center Amsterdam, Vrije Universiteit Amsterdam, Amsterdam Neuroscience, Amsterdam UMC location VUmc, Amsterdam, The Netherlands; Anatomy and Neurosciences, MS Center Amsterdam, Vrije Universiteit Amsterdam, Amsterdam Neuroscience, Amsterdam UMC location VUmc, Amsterdam, The Netherlands; Anatomy and Neurosciences, MS Center Amsterdam, Vrije Universiteit Amsterdam, Amsterdam Neuroscience, Amsterdam UMC location VUmc, Amsterdam, The Netherlands; Anatomy and Neurosciences, MS Center Amsterdam, Vrije Universiteit Amsterdam, Amsterdam Neuroscience, Amsterdam UMC location VUmc, Amsterdam, The Netherlands; Anatomy and Neurosciences, MS Center Amsterdam, Vrije Universiteit Amsterdam, Amsterdam Neuroscience, Amsterdam UMC location VUmc, Amsterdam, The Netherlands/Health, Medical and Neuropsychology Unit, Institute of Psychology, Leiden University, Leiden, The Netherlands; Neurology, MS center Amsterdam, Vrije Universiteit Amsterdam, Amsterdam Neuroscience, Amsterdam UMC location VUmc, Amsterdam, The Netherlands/Clinical Neurophysiology and MEG Center, MS Center Amsterdam, Vrije Universiteit Amsterdam, Amsterdam Neuroscience, Amsterdam UMC location VUmc, Amsterdam, The Netherlands; Anatomy and Neurosciences, MS Center Amsterdam, Vrije Universiteit Amsterdam, Amsterdam Neuroscience, Amsterdam UMC location VUmc, Amsterdam, The Netherlands; Anatomy and Neurosciences, MS Center Amsterdam, Vrije Universiteit Amsterdam, Amsterdam Neuroscience, Amsterdam UMC location VUmc, Amsterdam, The Netherlands

**Keywords:** Multiple sclerosis, inhibitory synaptic loss, network function; histopathology, computational modeling

## Abstract

**Background:**

Synaptic and neuronal loss contribute to network dysfunction and disability in multiple sclerosis (MS). However, it is unknown whether excitatory or inhibitory synapses and neurons are more vulnerable and how their losses impact network functioning.

**Objective:**

To quantify excitatory and inhibitory synapses and neurons and to investigate how synaptic loss affects network functioning through computational modeling.

**Methods:**

Using immunofluorescent staining and confocal microscopy, densities of glutamatergic and GABAergic synapses and neurons were compared between post-mortem MS and non-neurological control cases. Then, a corticothalamic biophysical model was employed to study how MS-induced excitatory and inhibitory synaptic loss affect network functioning.

**Results:**

In layer VI of normal-appearing MS cortex, excitatory and inhibitory synaptic densities were significantly lower than controls (reductions up to 14.9%), but demyelinated cortex showed larger losses of inhibitory synapses (29%). In our computational model, reducing inhibitory synapses impacted the network most, leading to a disinhibitory increase in neuronal activity and connectivity.

**Conclusion:**

In MS, excitatory and inhibitory synaptic losses were observed, predominantly for inhibitory synapses in demyelinated cortex. Inhibitory synaptic loss affected network functioning most, leading to increased neuronal activity and connectivity. As network disinhibition relates to cognitive impairment, inhibitory synaptic loss seems particularly relevant in MS.

## Introduction

Multiple sclerosis (MS) is a chronic inflammatory, demyelinating, and neurodegenerative disease of the central nervous system.^1^ In most patients, the disease progresses from an initial relapsing–remitting phase to a secondary-progressive phase characterized by gradual clinical worsening and progressive neurodegeneration.^2^ Two principal pathological hallmarks of this progressive phase are neuronal and synaptic loss, which are both related to clinical and cognitive functioning.^3,4^

Despite the frequent reports of neuronal and synaptic loss in MS, a hitherto undecided debate concerns the question whether excitatory or, rather, inhibitory neurons and synapses are more susceptible to MS pathology. Whereas some studies have purported that certain types of excitatory neurons are more vulnerable in MS,^5^ others have argued for a selective vulnerability of inhibitory cells.^6^ Also regarding excitatory and inhibitory synaptic loss, no definite conclusion has been reached which type, if any, occurs more in MS. Two recent studies found loss of inhibitory, but not excitatory, synapses in post-mortem MS hippocampi.^6,7^ Another study, however, found equal losses of both synaptic types.^8^ This debate is particularly urgent as synapses form the basis for neural communication and higher order network function, which is governed by a strict balance between excitation and inhibition.^4,9^ Recent work has shown that inhibitory synapses are especially important for cortical function and neural computation, despite their lower number.^10^ Healthy network dynamics are crucial for brain functioning and synaptic loss may perturb this, thus contributing to the network dysfunction and clinical impairment frequently seen in MS.^4,11^ These results warrant further quantifications of excitatory and inhibitory synaptic loss in MS, as well as investigations into the resulting effects on cortical network functioning.

Here, we aimed to achieve both these objectives. First, we determined whether excitatory or inhibitory neurons and synapses are more vulnerable to MS pathology using a large post-mortem MS dataset and detailed histopathological analysis. Second, the synaptic densities found in MS were used as input for an established corticothalamic biophysical model that accurately mimics large-scale brain networks.^12,13^ This allowed us to evaluate the differential and combined effects of excitatory and inhibitory synaptic loss on network functioning.

## Materials and methods

### Subjects and tissue collection

In this study, 33 cases with clinically definite and neuropathologically verified MS and 9 pathologically confirmed non-neurological controls (NCs) were included shortly after death. Tissue from the right superior frontal cortex was collected in a standardized manner by an experienced pathologist (supplemental methods). Tissue collection was done in collaboration with the Netherlands Brain Bank (NBB; http://www.brainbank.nl) and Normal Aging Brain Collection Amsterdam (NABCA; http://www.nabca.eu). This study complies with the institutional ethics guidelines. Subjects or their next of kin provided written informed consent for the use of their tissue and clinical information for research purposes to the NBB or NABCA.

### Histopathology

#### Staining protocol: cortical demyelination and region of interest selection

Stainings were performed on consecutive 10-µm-thick formalin-fixed and paraffin-embedded sections. Tissue sections were stained for myelin using proteolipid protein antibody to localize demyelinated regions. For subsequent analyses, uniform (i.e. six-layered) superior frontal cortex was selected to minimize cytoarchitecture heterogeneity and to avoid over- or underestimation of pathology in sulci and gyri, respectively. Areas were classified as demyelinated when clear demyelination was visible within the area, or otherwise as normal-appearing gray matter (NAGM). These selected regions of interest (ROIs) were randomized and two regions per participant were analyzed further. All stains and quantifications described below were performed within these same areas on consecutive sections and all analyses were performed in ImageJ (version 1.52a; https://imagej.net/Fiji). For details on the staining protocol for myelin, parvalbumin- (PV^+^) and calretinin-expressing (CR^+^) interneurons, NeuN-expressing neurons (NeuN^+^), and excitatory and inhibitory synapses, please see the supplemental methods.

#### Quantification of PV^+^, CR^+^, and NeuN^+^ neurons

Consecutive images at 20× magnification were taken with a multispectral imaging whole slide scanner (Vectra Polaris, PerkinElmer, USA) until the entire section was imaged. The “Analyze Particles” tool was used to perform a particle count within the ROIs across all cortical layers. In case of matching tissue types in both ROIs (i.e. both contained normal-appearing or demyelinated cortex), the results of the neuron counts were averaged and converted to counts/mm^2^. Outcome measures were thus densities of PV^+^, CR^+^, and NeuN^+^ neurons across all cortical layers.

#### Quantification of inhibitory and excitatory synapses

Sections of inhibitory (vGAT/gephyrin) and excitatory (vGLUT/PSD95) synapses were imaged using a confocal microscope (Nikon Instruments A1 Confocal Laser Microscope; Nikon Instruments Inc., USA). For this study, cortical layers I–III and VI were a priori included given their common demyelination in MS. Layers I–III are in close proximity to the cerebrospinal fluid (CSF) and most frequently involved in subpial lesions. Layer VI, being adjacent to the white matter (WM), is especially involved in leukocortical lesions. Therefore, images were taken in layers I–VI of the cortex at 1800× magnification. Layers were distinguished based on densities of pyramidal neurons. The “Synapse Counter” plugin was then used to determine co-localizations of pre- and post-synapses to obtain the count of “functional” synapses. Again, in case of matching tissue types in both ROIs, the synapse counts were averaged per cortical layer. Synaptic outcomes were counts/mm^2^ for each cortical layer (supplemental methods).

### In silico analysis: corticothalamic biophysical model

In order to evaluate the effects of altered excitatory and inhibitory synaptic densities on network functioning, an established corticothalamic mean-field model was employed. This biophysical model generates time series reflecting neuronal oscillations from magnetoencephalography (MEG) registrations (supplemental methods and previous studies).^12–14^ Because it is one of the few biophysical models that include the thalamus, it allows the generation of realistic alpha-band oscillations (8–13 Hz) via thalamo-cortical loops. The alpha band has previously been found to show clinically relevant alterations in MS.^15,16^ Moreover, this model allows the modulation of synaptic densities, making it especially suited to test the loss of synapses in MS. In short, the model is built up of units, each composed of two cortical (i.e. excitatory and inhibitory) and two thalamic populations. For each unit containing these four populations, excitatory and inhibitory synaptic connections exist between populations (Figure 3(a)). We allow for communication between brain regions by connecting the excitatory cortical connections between regions. The number of regions was chosen to reflect the 78 cortical regions of the Automated Anatomical Labeling atlas, a frequently used atlas in neuroimaging, and the structural connection strength between regions was based on empirical structural tractography data (Supplemental methods).

The computational model generates oscillations for each of the 78 brain regions. Equations and values used for all settings (except for synaptic densities and number of iterations) were purposefully kept identical to previously published work,^13^ in order to specifically study the effect of synaptic loss. Hence, each “run” consisted of 50 iterations in which synaptic densities were gradually reduced. The model was used for three conditions: first, to study the effects of excitatory synaptic loss, then inhibitory synaptic loss, and finally both synaptic types concurrently. Over the iterations respective synaptic types were reduced in a step-wise manner until one region reached a reduction equal to the empirical data of the post-mortem study (Figure 3 and Supplemental methods).

#### Biophysical model outcome measures

Outcome measures were chosen that reflect neuronal activity and functional connectivity (FC). For neuronal activity, we calculated the mean activity, defined as the power spectral density within the alpha band. For FC, the phase locking value (PLV) was calculated.^17^ When two signals “lock” phases (i.e. their phase difference is constant), they are assumed to have high connectivity. The PLV was calculated between all region pairs resulting in a weighted connectivity matrix which was averaged to obtain a mean PLV value for each iteration of the model.

#### Statistical analysis

Statistical analyses were performed using IBM SPSS26 (IBM Corp., USA). Demographics and post-mortem delay were compared between MS patients and controls using chi-square and Mann–Whitney *U* tests.

Neuronal (i.e. PV^+^, CR^+^, and NeuN^+^) and synaptic (i.e. excitatory and inhibitory) densities were compared using linear mixed-effects models, to account for nested data (i.e. multiple ROIs and, for synapses, multiple cortical layers per individual). Fixed effects consisted of tissue type (i.e. NC cortex, MS NAGM, and MS demyelinated cortex) and, for synapses, cortical layer (i.e. I–VI). Analyses were controlled for age, sex, and post-mortem delay. Outcomes of interest for neurons were differences between tissue type and, for synapses, interactions between tissue type and layer. In case of significant interaction effects in the synapse analyses (i.e. *p* values < 0.05), linear mixed-effects models were applied for each layer separately. Residuals were checked for normality.

## Results

### Demographics

Demographic and neuropathological data are displayed in Table 1 and Supplemental Table S1. Whereas MS and NC groups did not differ on age and sex distribution, post-mortem delay was significantly shorter in patients (*p* < 0.001).

**Table 1.** Demographic characteristics.

	NC (*N* = 9)	MS (*N* = 33)	*p* value
Female (%)	5 (55.6)	21 (63.6)	0.711
Age (years)	72 (70–77.5)	60 (55–75.5)	0.065
Post-mortem delay (minutes)	600 (405–700)	315 (255–382.5)	**<0.001**

Values are presented as median (IQR) unless specified otherwise.

## Neuronal densities

**Figure 1.**
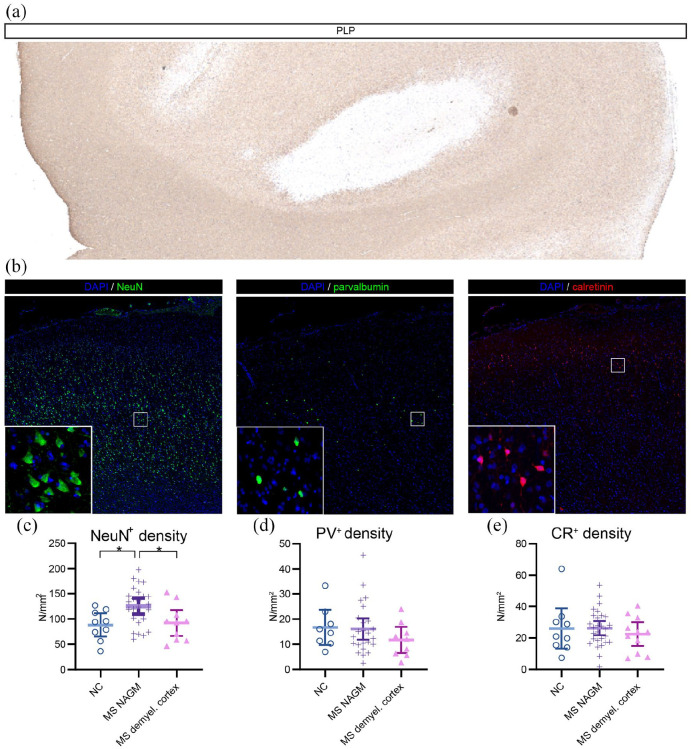
Myelin and neuronal immunohistopathological stainings and quantifications: (a) Myelin staining using PLP showing demyelinated MS cortex, (b) Stainings for NeuN-expressing neurons (NeuN^+^), parvalbumin-expressing interneurons (PV^+^), and calretinin-expressing interneurons (CR^+^) showing all cortical layers. (c) Quantifications of neuronal types for non-neurological control cortex (NC), multiple sclerosis normal-appearing gray matter (MS NAGM), and multiple sclerosis demyelinated cortex (MS demyel. cortex) with means and 95% confidence intervals.

Of the 42 (33 MS and 9 NC) tissue blocks, a total of 88 ROIs (71 MS, 17 NC) were analyzed. In MS tissue, 17/71 (23.9%) ROIs were demyelinated, and the remaining 54 (76.1%) were NAGM (Figure 1(a)). All NC ROIs were normal gray matter. After averaging the ROIs that were classified as the same type, a total of 9 NC, 29 MS NAGM, and 13 MS demyelinated cortex measurements were entered in the final analysis. PV^+^- and CR^+^-interneuron densities did not differ between tissue types (Figure 1(b), (d), and (e); Table S1), but NeuN^+^ neuronal densities did show significant differences (*F*(2, 38) = 4.472, *p* = 0.018). Post hoc tests showed NeuN^+^ densities to be higher in MS NAGM compared to NC (*p* = 0.038) and MS demyelinated cortex (*p* = 0.015; Figure 1(b) and (c)). As the increased NeuN^+^ densities in MS NAGM could be caused by tissue compaction, this may have influenced the interneuron results. To minimize this effect, we repeated the interneuron analysis with NeuN^+^ density as covariate. This did not alter the results.

## Synaptic densities

**Figure 2.**
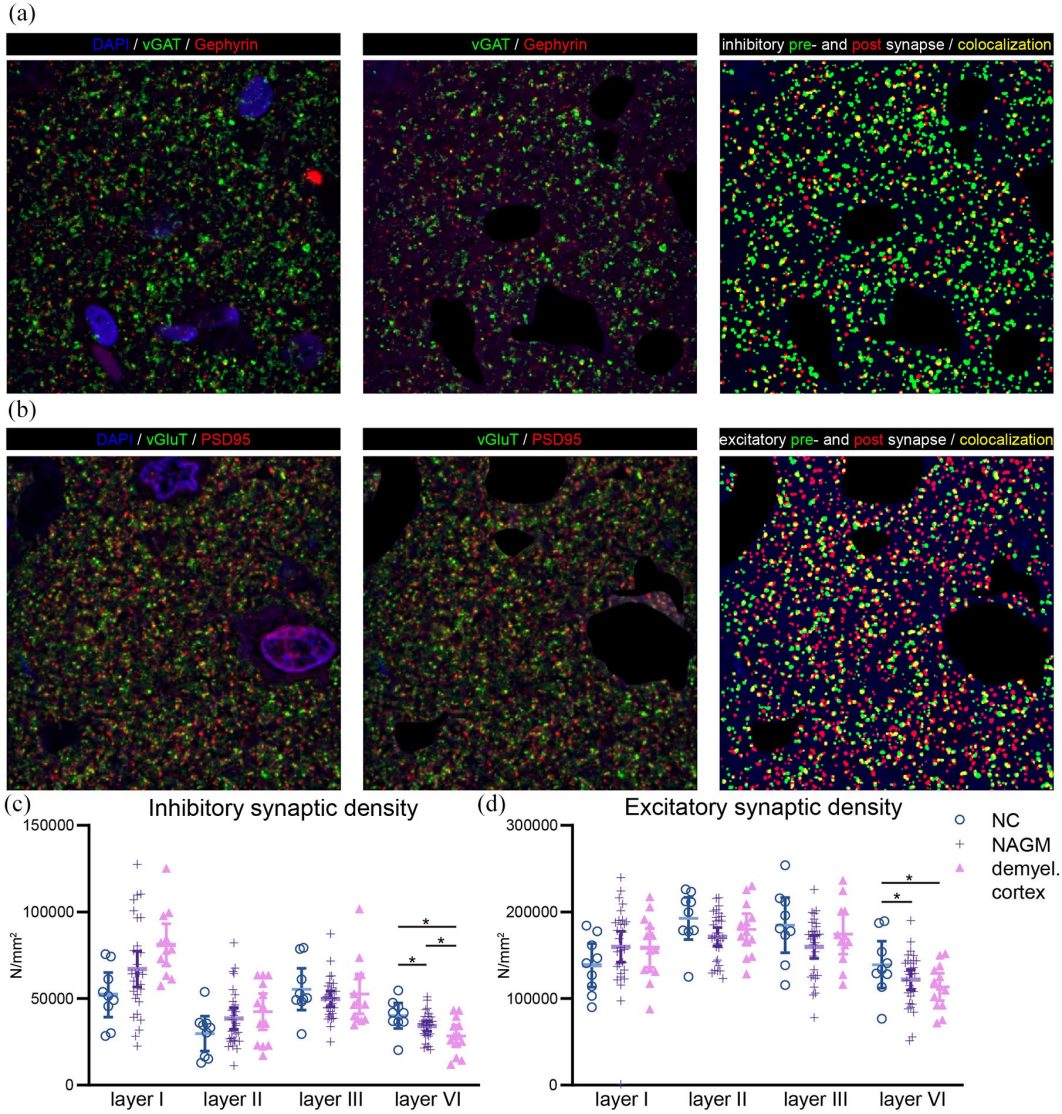
Immunofluorescent stainings and quantifications of inhibitory and excitatory synapses. (a) Inhibitory presynaptic vesicular GABA transporter (vGAT, in green), postsynaptic inhibitory protein gephyrin (in red), and a merged image in which yellow indicates co-localization of inhibitory pre- and postsynaptic elements. Nuclei were stained using 4′,6-diamino-2-phenylindole (DAPI, in blue). (b) Excitatory presynaptic vesicular glutamate transporter (vGluT in green), postsynaptic excitatory protein PSD95 (in red), and a merged image in which yellow indicates co-localization of excitatory pre- and postsynaptic elements. (c) Quantifications of inhibitory and excitatory synaptic densities across layers I, II, III, and VI for the three tissue types. NC: non-neurological control cortex; NAGM: multiple sclerosis normal-appearing gray matter; demyel. cortex: multiple sclerosis demyelinated cortex.

For both excitatory (*F*(6, 150.15) = 3.13, *p* = 0.006) and inhibitory (*F*(6, 152.97) = 3.68, *p* = 0.002) synaptic densities, a significant interaction effect was observed between tissue type and cortical layer. Post hoc analyses revealed an increased excitatory synaptic density in cortical layer I (*p* = 0.049) and a reduction of both synaptic types in cortical layer VI (excitatory: *p* = 0.004; inhibitory: *p* *=* 0.002, Figure 2, Table S2). However, after repeating this analysis with NeuN^+^ density as covariate to correct for potential tissue compaction, only layer VI remained significant (supplemental results). In layer VI, both synaptic types were reduced in MS NAGM and demyelinated cortex as compared to NC (*excitatory*: MS NAGM vs. NC: −12.5%, *p* = 0.003, demyelinated cortex vs. NC: −18.5%, *p* = 0.001; *inhibitory*: MS NAGM vs. NC: −14.9%, *p* = 0.039, demyelinated cortex vs. NC: −29.3%, *p* = 0.001, demyelinated cortex vs. NAGM: −14.4%, *p* = 0.037, Figure 2(c) and (d)**)**. Thus, reductions in layer VI MS NAGM of excitatory and inhibitory synapses compared to NC values were similar (i.e. −12.5% and −14.9%, respectively). But, for demyelinated cortex, the reduction was significantly larger for inhibitory than for excitatory synapses (i.e. −29.3% vs. −18.5%, *p* = 0.047).

## Corticothalamic biophysical model

**Figure 3.**
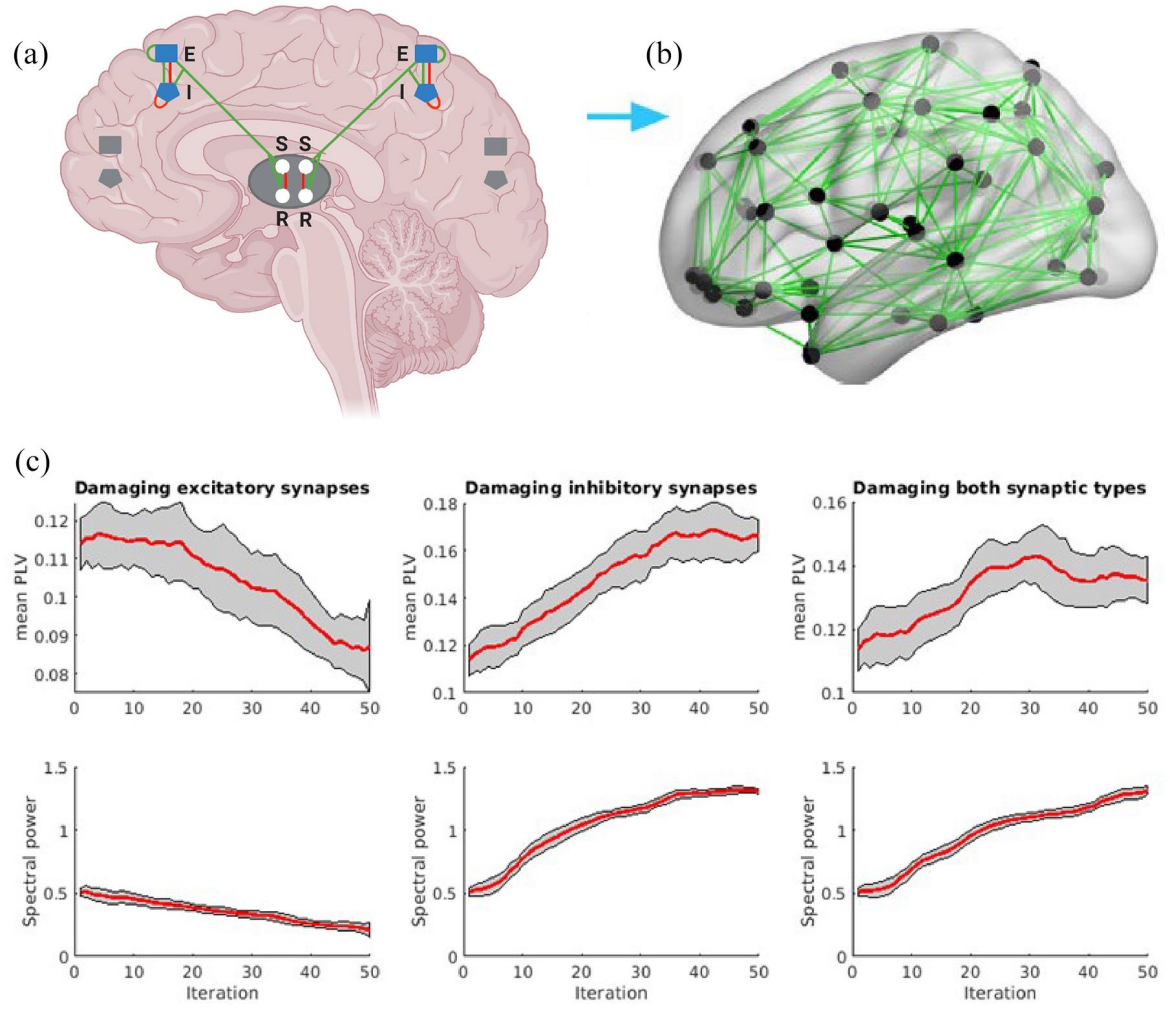
Overview of biophysical model and functional consequences of synaptic loss. (a) Each cortical region is composed of units consisting of neural populations with coupled excitatory (E) and inhibitory (I) cortical and thalamic reticular (R) and relay (S) components. (b) The empirically informed white matter connections are displayed. Figure was adapted from Tewarie et al.^13^ and Figure (a) was created with BioRender.com. (c) The effect of synaptic loss on functional connectivity (mean PLV, top row) and neuronal activity (spectral power, bottom row). Shown are the mean values over iterations (red lines) with standard deviations (gray areas). It can be appreciated that despite similar relative reductions in densities for excitatory and inhibitory synapses, damage to both synaptic types leads to an increase in both functional connectivity and neuronal activation.

For the network analyses, the levels of excitatory and inhibitory synaptic loss as found in normal-appearing MS cortical layer VI were set as model end-points, as this layer showed significantly altered synaptic densities. Thus, in 50 iterations, the excitatory and inhibitory synapses were gradually reduced until their respective end points (i.e. −12.5% for excitatory and −14.9% for inhibitory synapses). Figure 3(c) shows the change in alpha power and PLV for the three model conditions in which excitatory, inhibitory, and both synaptic types combined were reduced from empirical control levels toward MS densities. At the 50th iteration (i.e. MS level), excitatory synapse loss led to a monotonic decrease for both the alpha power (−59.0% relative to initial conditions) and PLV (−23.4% relative to initial conditions). Conversely, reducing inhibitory synapses resulted in an opposite, stronger effect, that is, an increase for both the alpha power and PLV that reached a plateau after approximately 35 iterations and resulted in an increase in alpha power of +156.6% relative to the initial conditions and PLV of +46.3% after 50 iterations. Reducing both synaptic types simultaneously resulted in a pattern very similar to only reducing inhibitory synapses: an increase for both the alpha power (+158.1% relative to initial conditions) and PLV (+19.0%).

## Discussion

The current study investigated whether excitatory or inhibitory neurons and synapses are more vulnerable to MS pathology and how excitatory and inhibitory synaptic loss affect network function. Post-mortem MS cortical layer VI showed reductions in both excitatory and inhibitory synaptic densities. In MS NAGM layer VI, excitatory and inhibitory synaptic losses compared to NC were −12.5% and −14.9%, respectively. In MS demyelinated cortex layer VI, inhibitory synaptic loss was more severe, as this reached levels of −29.3% versus −18.5% for excitatory synapses. In our computational model, reducing excitatory synapses led to a decrease in both neuronal activity (i.e. alpha-band spectral power) and FC (i.e. PLV), whereas reducing inhibitory synapses showed the opposite: an increase in both measures. Interestingly, when reducing both synaptic types simultaneously, a disinhibitory increase was observed in both neuronal activity and FC. Thus, despite the fact that MS affects both excitatory and inhibitory synapses, inhibitory synaptic loss was found to affect network function more severely.

This is one of the first studies translating damage at the synaptic level to macroscopic functional changes. Using histopathology and computational modeling, we show that even minor changes in synaptic density can lead to large network shifts. Computational models have the ability to connect data across levels of experimentation and the current neural mass model specifically generates whole-brain neuronal dynamics based on summed action potentials of individual neuronal populations.^12,19^ This particular model has been shown to accurately mimic large-scale brain dynamics and to capture pathological brain states such as MS.^13,20^ In addition, this model includes the thalamus, which allows the generation of corticothalamic alpha oscillations. In MS, increased alpha-band power and connectivity and corticothalamic tract damage are correlates of clinical and cognitive dysfunction, making this neural mass model the optimal choice for the current analysis.^15,21^ Here, we extend previous studies by feeding real-world excitatory and inhibitory synaptic densities into the model. Our results not only corroborate previous work that found inhibitory synaptic functioning to be of particular importance for cortical function and cognition but they also suggest that the levels of synaptic loss seen in end-stage MS substantially perturb this dynamic.^10^

The PV^+^- and CR^+^-interneuron densities did not differ between groups. Although some earlier studies did report loss of these neuronal types in MS, unchanged densities have also been found.^5,6,22^ These varying reports could be due to variations in the investigated brain regions or cortical layers, or could be due to the relatively low number of interneurons.^23^ By contrast, NeuN^+^-expressing neuron densities were higher in MS NAGM compared to NC and MS demyelinated cortex. Despite neuronal loss being a pathological hallmark of MS, these results are not completely unexpected. Equal or even higher neuronal densities have been found previously in MS and have been explained by tissue compaction.^8,24,25^ In our MS NAGM sample, there may have been a limited amount of neuronal loss that hypothetically coincided with substantial tissue compaction, which may then have resulted in higher perceived neuronal densities. In MS demyelinated cortex, it could be that both neuronal loss and tissue compaction are present due to higher levels of demyelination and resulted in a net neuronal density similar to NC cortex. However, as additional measures such as glial densities were out of scope for the current study, this explanation remains tentative.

Synaptic loss was only noted in cortical layer VI, which may seem surprising as previous literature suggests that superficial layers are more affected, potentially due to meningeal infiltrates or immune mediators in the CSF.^26^ Cortical layer VI projects mainly to the thalamus, forming modulatory feedback loops.^27^ This may explain why synaptic loss was observed solely in this layer, as the thalamus shows early and severe structural disconnection and atrophy in MS.^28^ As a result of retrograde degeneration, it is possible that inflammation and corticothalamic disconnection induce distant synaptic loss in cortical layer VI.^18^ In MS, synapses are engulfed and stripped by microglia and macrophages.^29,30^ This is considered neuroprotective in the healthy brain, but is thought to go astray in MS. Increased synaptic stripping is likely caused by complement deposition in synaptic terminals, which primes the synapses for phagocytosis, and which may predominantly target inhibitory synapses.^7,2930[Bibr bibr31-13524585221125381]31^ These studies, in combination with work suggesting that synaptic loss may be reversible with treatment, may provide new avenues to understand and, eventually, prevent synaptic loss in MS.^3,32^

The synaptic loss and network disinhibition that were observed have clinical implications as well. MEG and functional magnetic resonance imaging (fMRI) studies have observed increased alpha power and corticothalamic connectivity in MS and found that this related to cognitive dysfunction.^15,33,34^ This suggests that the hyperconnectivity often seen in MS could be the result of inhibitory synaptic loss and implies that this is not a process of functional reorganization, but rather maladaptive disinhibition, which could also be related to epilepsy seen frequently in MS.^35^ As the thalamus is a hub in the brain network and pivotal for cognition, MS-induced thalamic atrophy and increased corticothalamic signaling may eventually lead to cognitive deterioration. However, due to our post-mortem study design, it is not yet possible to make direct generalizations to the MS population.

This work is not without limitations. First, post-mortem delay was significantly shorter in MS than in NC, whereas NCs were several years older (*p* = 0.065). Therefore, all analyses were corrected for age and post-mortem delay. Second, in the histopathological analysis, only the right superior frontal cortex layers I–III and VI were studied. As neuronal and synaptic loss may fluctuate between brain regions, this limits the generalizability of our results. In addition, we a priori selected cortical layers I–III and VI due to their common demyelination in MS, but this does not exclude the possibility that layers IV and V have altered neuronal and synaptic densities as well. Furthermore, superior frontal cortex cytoarchitecture may have been heterogeneous and could have influenced our results. However, this was minimized by our standardized dissection protocol and by only selecting straight, uniform cortex. Next, using a z-stack in which pre- and postsynaptic co-localizations were maximum projected to obtain a two-dimensional image was suboptimal. Future studies should include more regions and use a three-dimensional slab. The neural mass model that was employed has been shown to accurately reflect healthy and pathological brain states,^12–14^ but cannot be said to have captured a multidimensional disease such as MS in its entirety. Here, we have deliberately chosen to only model excitatory and inhibitory synaptic loss due to relevance of these synaptic types for network functioning. But, future work should also take into account individual cortical layers, white and gray matter loss, and specifically incorporate thalamic histopathology data, which was not possible for the current study.^13^

In conclusion, by translating from micro- to macro-scale levels of experimentation, this study shows how comparable losses of excitatory and inhibitory synapses in MS may have diverging effects on macro-scale networks. In our computational model, the loss of inhibitory synapses impacted the network more profoundly, leading to a disinhibitory increase in FC (PLV) and neuronal activity (alpha power). As both these changes in the alpha band are known to relate to cognitive impairment in MS, our results stress that inhibitory synaptic loss is of particular importance in MS-related network dysfunction.

## Supplemental Material

sj-docx-1-msj-10.1177_13524585221125381 – Supplemental material for Inhibitory synaptic loss drives network changes in multiple sclerosis: An ex vivo to in silico translational study Click here for additional data file. Supplemental material, sj-docx-1-msj-10.1177_13524585221125381 for Inhibitory synaptic loss drives network changes in multiple sclerosis: An ex vivo to in silico translational study by Marijn Huiskamp, Svenja Kiljan, Shanna Kulik, Maarteen E Witte, Laura E Jonkman, John GJM Bol, Geert J Schenk, Hanneke E Hulst, Prejaas Tewarie, Menno M Schoonheim and Jeroen JG Geurts in Multiple Sclerosis Journal
