# Supplementary material for: Inhibitory synaptic loss drives network changes in multiple sclerosis: An ex vivo to in silico translational study
Source: Mult Scler. 2022 Oct 3;28(13):2010–9. doi: 10.1177/13524585221125381 (PMC9574900; doi:10.1177/13524585221125381)
Supplement: sj-docx-1-msj-10.1177_13524585221125381 – Supplemental material for Inhibitory synaptic loss drives network changes in multiple sclerosis: An ex vivo to in silico translational study [file sj-docx-1-msj-10.1177_13524585221125381.docx]

**Supplemental methods**

*Staining protocol: cortical demyelination*

Tissue collection was performed according to a standardized protocol and samples from the superior frontal gyrus were selected on the first coronal slice where the frontal horns of the lateral ventricles were identified.^1,2^ For the NCs, strict inclusion criteria were used and their non-neurological status was confirmed by an experienced pathologist.^2^ Subsequently, sections were stained for myelin to localize demyelinated regions using proteolipid protein antibody (PLP; 1:500; mouse anti bovine; MCA839G; Bio-Rad Laboratories B.V., Hercules, CA, USA. Detection of the PLP antigen was visualized using 3,3-diaminobenzidine (DAB, Sigma, St. Louis, MO, USA) as a chromogen. Counter stain with Nissl was used to detect areas with uniform (i.e. six-layered, straight) cortex and to detect demyelination. Cortical layers were distinguished based on pyramidal cell density. Cortical layer I is located closest to the surface and contains very few pyramidal cells. Layer II is characterized by a larger number of small pyramidal cells, whereas layer III contains more pyramidal cells of medium size. Layer IV is relatively thin and also contains pyramidal cell bodies. Layer V contains larger pyramidal cell bodies and finally, layer VI is located closest to the WM.^3^

*Staining protocol: Parvalbumin-, calretinin- and NeuN-expressing neurons*

Sections were deparaffinized in xylene and rehydrated in graded series of ethanol and rinsed in Milli-Q water. Next antigen retrieval was performed in a pre-heated Tris-EDTA buffer, 10mM, pH9, in a steam cooker for 35 minutes. Afterwards sections were left in the same buffer to cool down to room temperature (RT). Then sections were rinsed in tris-buffered saline (TBS) and subsequently incubated with blocking solution (3% normal donkey serum in TBS with 0.1% triton) to prevent non-specific binding. After these steps sections were incubated with primary antibodies in blocking solution over night at 4°C (parvalbumin, PV^+^; 1:200; rabbit anti rat; PV-27; Swant, Fribourg, Switzerland; calretinin, CR^+^; 1:200; goat anti rat; AB1550; Chemicon, Darmstadt, Germany; and NeuN^+^; 1:400; rabbit anti rat; NBP1-77686; Novus Biologicals, Centennial, CO, USA). The next day sections were rinsed 3x 5 minutes in TBS. Secondary antibodies were added in blocking solution and incubated for 1.5h at RT for PV^+^ and CR^+^ and 2h for NeuN^+^ (for PV^+^ and NeuN^+^: donkey anti rabbit Alexa594; 1:200; A21207; Invitrogen Molecular Probes, Bar Harbor, ME, USA; for CR^+^: donkey anti goat Alexa647; 1:200; A21447; Invitrogen Molecular Probes, Bar Harbor, ME, USA). Then sections were rinsed 3x 5 minutes in TBS. 4’,6-diamino-2-phenylindole (DAPI) staining (1:1000; Sigma, St. Louis, MO, USA) was applied for 5 minutes and rinsed with TBS afterwards. Finally, sections were coverslipped with Mowiol.

*Staining protocol: Excitatory and inhibitory synapses*

Deparaffination, antigen retrieval and blocking were performed as described in the previous section. Then for the excitatory synapse fluorescent double staining, pre-treated sections were incubated with vesicular glutamate transporter (vGLUT) antibody in blocking solution (vGLUT; 1:400; guineapig anti rat; 135304; Synaptic Systems, Goettingen, Germany). For the inhibitory synapse fluorescent double staining, pre-treated sections were incubated with vesicular GABA transporter (vGAT) antibody in blocking solution (vGAT; 1:100; chicken anti rat; 131 006; Synaptic Systems, Goettingen, Germany). Both were incubated for 3h at RT and afterwards overnight at 4°C. The next day sections were rinsed 3x 5 minutes in TBS. Afterwards for the excitatory synapse fluorescent double staining, pre-treated sections were incubated with postsynaptic density protein 95 (PSD95) antibody in blocking solution (PSD95; 1:150; rabbit anti human; #3450S; Cell Signaling Technology, Inc., Danvers, MA, USA). For the inhibitory synapse fluorescent double staining gephyrin antibody in blocking solution was added to the sections (gephyrin; 1:100; mouse anti rat; 147021; Synaptic Systems, Goettingen, Germany). Both stainings were first incubated 4h at RT and afterwards for two nights overnight at 4°C. Then sections were rinsed 3x 5 minutes in TBS. Secondary antibodies were added in blocking solution and incubated for 3h at RT. For the excitatory synapse staining these were (donkey anti guineapig alexa 488; 1:200; 706-545-148; The Jackson Laboratory, Bar Harbor, ME, USA; and donkey anti rabbit alexa 546; 1:200; A10040; Invitrogen, Bar Harbor, ME, USA). For the inhibitory synapse staining these were (donkey anti chicken alexa 488; 1:200; 703-545-155; The Jackson Laboratory, Bar Harbor, ME, USA; and donkey anti mouse alexa 546; 1:200 A10036; Invitrogen Molecular Probes, Bar Harbor, ME, USA). Then sections were rinsed 3x 5 minutes in TBS. Sudan black was applied to the sections (0.1% in 70% ethanol) for 5 minutes, then rinsed in demi water. Finally, sections were coverslipped with Mowiol.

*Quantification of neurons and synapses*

Image analysis was performed in ImageJ (version 1.52a, <https://imagej.net/Fiji>).^4^ For PV^+^, CR^+^ and NeuN^+^ images the two pre-determined ROIs per section were identified and manually drawn in every image. Then, the different neuronal types were automatically segmented and images were binarized. For PV^+^ and CR^+^ images, one threshold was chosen for all images. Due to inter-individual variation in NeuN^+^ staining intensity, individual thresholds were chosen for each NeuN+ image with optimal sensitivity to neurons and minimal background signal. The quantifications were performed by two investigators (S. Kiljan and MH) blinded for tissue type. For excitatory and inhibitory synapses, images were taken in layer 1, 2, 3 and 6 of the cortex at 1800x magnification. A z-stack was obtained of 2µm, with step size of 0.1µm. The ten middle stack slices were selected, comprising a total of 1µm thickness. Subsequently, these slices were maximum projected to obtain a two-dimensional image. The “Synapse Counter” plugin was used to determine the synapse count.^5^ After maximum projecting, DAPI^+^ nuclei were manually outlined in ImageJ, their area was measured, and they were removed from the image. Finally, the synaptic density was calculated from the number of synapses identified divided by the image area minus the nucleic area. Segmentations were visually inspected independently by S. Kiljan and MH who were blinded for tissue type and when both investigators agreed that the segmentation was not representative for the original raw data (e.g. when the image was overexposed or segmentation did not match the original image) it was excluded from analyses.

**In silico analysis: biophysical model parameters**

As stated in the main text, the model is built up of units, each composed of two cortical (i.e. excitatory and inhibitory) and two thalamic (i.e. relay nucleus and reticular nucleus) populations (see fig. 3A, B). For each unit containing these four populations, the mean synaptic membrane potential is modulated by the firing rate of other populations in the unit and by the firing rate of the population itself. Here, firing rate can be considered as the aggregation of incoming action potentials. In turn, the mean synaptic membrane potential of a population is translated into a firing rate (occurring at the soma of the neurons).^6–8^ We allow for communication between brain regions by connecting the excitatory cortical connections between regions. This cortico-cortical coupling is tuned by the so-called coupling strength. We further define a noise parameter to capture stochastic physiological variation.^9^ As the quantified synaptic densities were measured in the cortex, we modulated the cortical excitatory and inhibitory synaptic populations projecting onto itself and onto each other (i.e. E🡪E, I🡪I, E🡪I and I🡪E). The number of regions was chosen to reflect the 78 cortical regions of the Automated Anatomical Labeling atlas^10^, a frequently used atlas in neuroimaging, and the structural connection strength between regions was based on empirical structural tractography data.^11^

**In silico analysis: mimicking MS pathology**

In order to generate whole-brain functional data, MEG data were simulated from the oscillations of all 78 regions. Therefore, the synaptic data from the superior frontal gyrus were extrapolated to the other AAL regions. In other words, all 78 regions were given a certain probability to have their synaptic density reduced, with a maximum reduction equal to what was empirically observed in the superior frontal cortex. However, that probability was not equal for every region, as described below. Since cortico-thalamic loops are central in this model and white matter tracts running between the thalamus and cortex are known predilection sites for MS lesions, real-world MS cortico-thalamic white matter integrity data was used to constrain the damage patterns in the model.^12^ More specifically, we extracted the percentage of damage in each corticothalamic tract as found in a MS dataset of 123 patients who underwent diffusion weighted MR imaging and probabilistic tractography.^13^ Grey matter regions connected to the thalamus via a more heavily damaged tract were assigned a higher probability to become damaged in the model. Thus, the probability for each region’s synaptic densities to be reduced was not equal, but was proportional to the damage in the tract running from that region to the thalamus, in order to mimic MS pathology more accurately while being sensitive to the measure that was of interest the most: synaptic loss. In order to optimally compare the effects of excitatory and inhibitory synaptic loss, the distributions of damage across regions for the separate conditions of the model were kept identical (i.e. for each run with excitatory, inhibitory and combined synaptic loss, the regions in which synaptic densities were reduced on each iteration were the same). Finally, as the model does not distinguish between cortical layers, the synaptic densities were entered in the model from those cortical layers that showed significant deviations from NC data after correction for NeuN^+^-density (i.e. due to potential tissue compaction).

**Supplemental results**

**Table S1 | Demographic and neuropathology data of non-neurological controls (NC) and MS patients.**

| **Case** | **Sex** | **Age**  **(y)** | **PMD (hh:mm)** | **MS clinical phenotype** | **DD** | **Cause of death** |
| --- | --- | --- | --- | --- | --- | --- |
| **NC01** | F | 72 | 07:15 | **-** | **-** | Heart failure |
| **NC02** | F | 69 | 13:00 | **-** | **-** | Pulmonary embolism |
| **NC03** | M | 59 | 08:00 | **-** | **-** | Euthanasia |
| **NC04** | M | 72 | 11:45 | **-** | **-** | Metastatic esophagus cancer and lung failure |
| **NC05** | M | 77 | 12:00 | **-** | **-** | Pneumonia |
| **NC06** | F | 79 | 06:15 | **-** | **-** | Unknown |
| **NC07** | F | 78 | 10:00 | **-** | **-** | Unknown |
| **NC08** | F | 71 | 06:30 | **_-_** | **_-_** | Lung carcinoma |
| **NC09** | M | 74 | 10:15 | **_-_** | **_-_** | Euthanasia |
| **MS01** | F | 76 | 02:30 | PPMS | 23 | Pyelonephritis with palliative care |
| **MS02** | F | 35 | 04:00 | ND | 10 | Euthanasia |
| **MS03** | F | 74 | 03:30 | SPMS | 50 | Euthanasia |
| **MS04** | F | 59 | 05:10 | RRMS | 22 | Euthanasia |
| **MS05** | M | 54 | 04:15 | SPMS | 21 | Euthanasia |
| **MS06** | F | 57 | 02:30 | SPMS | 34 | Euthanasia |
| **MS07** | F | 50 | 04:40 | PPMS | 12 | Euthanasia |
| **MS08** | M | 84 | 03:10 | PPMS | ND (>20y) | Pneumonia |
| **MS09** | F | 47 | 06:25 | SPMS | 27 | Pneumonia |
| **MS10** | F | 73 | 02:40 | ND | 30 | Euthanasia |
| **MS11** | F | 59 | 06:00 | ND | 28 | Euthanasia |
| **MS12** | F | 52 | 03:30 | ND | 11 | Myocardial infarction |
| **MS13** | F | 77 | 02:40 | ND | 27 | Respiratory insufficiency due to pleuritic carcinomatosa, palliative sedation |
| **MS14** | F | 52 | 05:35 | SPMS | 16 | Pneumonia, palliative sedation |
| **MS15** | M | 75 | 05:05 | SPMS | 14 | Respiratory insufficiency, palliative sedation |
| **MS16** | M | 78 | 05:05 | SPMS | 40 | MS, CVA, dehydration, cachexia |
| **MS17** | M | 66 | 05:00 | SPMS | 27 | Possible pneumonia and/or congestive heart failure |
| **MS18** | M | 75 | 04:05 | PPMS | 42 | Euthanasia |
| **MS19** | M | 58 | 04:00 | SPMS | 27 | Pneumonia and terminal renal insufficiency |
| **MS20** | M | 71 | 04:00 | PMS | 15 | Lung carcinoma with osseous metastases |
| **MS21** | M | 59 | 05:00 | PMS | ND | Euthanasia |
| **MS22** | M | 60 | 02:00 | PMS | 31 | Respiratory failure due to pneumonia |
| **MS23** | F | 60 | 03:25 | PPMS | 11 | Progressive MS with decreased intake |
| **MS24** | M | 66 | 05:00 | PPMS | 25 | Euthanasia |
| **MS25** | F | 61 | 04:30 | SPMS | ND | Euthanasia |
| **MS26** | F | 57 | 04:00 | SPMS | 25 | Euthanasia |
| **MS27** | F | 70 | 03:00 | SPMS | 32 | Euthanasia |
| **MS28** | M | 81 | 05:00 | PPMS | 44 | Pneumonia |
| **MS29** | F | 77 | 03:00 | SPMS | 26 | CVA and dehydration |
| **MS30** | F | 82 | 03:40 | ND | ND | Euthanasia |
| **MS31** | F | 52 | 03:40 | ND | ND | Euthanasia |
| **MS32** | F | 39 | 02:30 | SPMS | 8 | Progressive MS, decreased intake and palliative sedation |
| **MS33** | F | 48 | 03:55 | SPMS | 24 | Pneumonia, palliative sedation |

PMD = post-mortem delay; DD = disease duration; ND = not determined; PPMS = primary progressive MS; SPMS = secondary progressive MS; PMS = progressive MS, meaning that the clinical course was progressive, but not possible to specify that further into primary or secondary progressive MS; CVA = cardiovascular accident.

**Table S2 | Neuronal and synaptic densities per tissue type.** Shown are N per group, mean density in counts/mm^2^ (SD), 95% confidence interval and percentage difference between MS tissue types and NC for significant comparisons.

| **Density** (counts/mm^2^) | **NC** | **MS NAGM** | **MS demyelinated cortex** | ***P*-value** |
| --- | --- | --- | --- | --- |
| **PV^+^ interneurons**   - Mean (SD) - 95% CI | - N = 8 - 16.7 (8.4) - 9.7-23.7 | - N = 24 - 16.6 (9.8) - 12.4-20.7 | - N = 9 - 11.7 (6.8) - 6.5-16.9 | 0.489 |
| **CR^+^ interneurons**   - Mean (SD) - 95% CI | - N = 9 - 26.0 (16.6) - 13.3-38.8 | - N = 29 - 26.1 (11.3) - 21.8-30.4 | - N = 11 - 21.7 (11.4) - 14.0-29.3 | 0.660 |
| **NeuN^+^ neurons**   - Mean (SD) - 95% CI - % diff. NC | - N = 9 - 88.0 (29.8) - 65.2-110.9 - - | - N = 25 - 125.2 (38.3) - 109.4-141.0 - +42.3% | - N = 10 - 92.1 (35.6) - 66.6-117.5 - +4.7% | **0.018^a^** |
| **E synapses all layers**   - Mean (SD)*10^4 - 95% CI*10^4 | - N = 36 - 16.4 (4.2) - 14.9-17.8 | - N = 115 - 15.4 (3.8) - 14.7-16.1 | - N = 51 - 15.6 (4.1) - 14.5-16.8 | **0.006^b^** |
| **I synapses all layers**   - Mean (SD)*10^4 - 95% CI *10^4 | - N = 36 - 4.4 (1.7) - 3.9-5.0 | - N = 116 - 4.74 (2.15) - 4.3-5.1 | - N = 52 - 4.98 (2.54) - 4.3-5.7 | **0.002^b^** |
| **E synapses L1**   - Mean (SD)*10^4 - 95% CI*10^4 | - N = 9 - 13.8 (3.3) - 11.3-16.3 | - N = 28 - 16.5 (3.6) - 15.1-17.9 | - N = 13 - 15.8 (3.7) - 13.6-18.1 | 0.049^c^ |
| **I synapses L1**   - Mean (SD)*10^4 - 95% CI*10^4 | - N = 9 - 5.2 (1.7) - 3.9-6.5 | - N = 29 - 6.7 (2.7) - 5.7-7.7 | - N = 13 - 7.6 (2.6) - 6.0-9.1 | 0.220 |
| **E synapses L2**   - Mean (SD)*10^4 - 95% CI*10^4 | - N = 9 - 19.2 (3.2) - 3.9-6.5 | - N = 29 - 17.1 (2.8) - 16.1-18.2 | - N = 13 - 18.0 (3.0) - 16.2-19.8 | 0.600 |
| **I synapses L2**   - Mean (SD)*10^4 - 95% CI*10^4 | - N = 9 - 3.0 (1.3) - 2.0-4.0 | - N = 29 - 3.8 (1.6) - 3.2-4.5 | - N = 13 - 4.2 (1.7) - 3.2-5.3 | 0.453 |
| **E synapses L3**   - Mean (SD)*10^4 - 95% CI*10^4 | - N = 9 - 18.5 (4.1) - 15.3-21.7 | - N = 29 - 16.0 (3.5) - 14.6-17.3 | - N = 12 - 17.4(3.6) - 15.1-19.7 | 0.155 |
| **I synapses L3**   - Mean (SD)*10^4 - 95% CI*10^4 | - N = 9 - 5.5 (1.6) - 4.3-6.7 | - N = 29 - 5.0 (1.2) - 4.5-5.4 | - N = 13 - 5.3 (1.9) - 4.1-6.4 | 0.286 |
| **E synapses L6**   - Mean (SD)*10^4 - 95% CI*10^4 - % diff. NC | - N = 9 - 13.9 (3.5) - 11.2-16.6 - - | - N = 29 - 12.2 (3.1) - 11.0-13.4 - -12.5% | - N = 13 - 11.3 (2.6) - 9.8-12.9 - -18.5% | **0.004^d^** |
| **I synapses L6**   - Mean (SD)*10^4 - 95% CI*10^4 - % diff. NC | - N = 9 - 4.0 (1.0) - 3.3-4.7 - - | - N = 29 - 3.4 (0.8) - 3.1-3.7 - -14.9% | - N = 13 - 2.8 (1.0) - 42.2-3.5 - -29.3% | **0.002^e^** |

^a^ NC vs. MS NAGM P = 0.038, NC vs. MS demyelinated cortex P = 0.015; ^b^ P-value pertains to group*layer interaction effect. ^c^ P-value after correction for NeuN^+^ density: 0.134. ^d^ NC vs. MS NAGM P = 0.003, NC vs. MS demyelinated cortex P = 0.001. ^e^ NC vs. MS NAGM P = 0.039, NC vs. MS demyelinated cortex P = 0.001, MS NAGM vs. demyelinated cortex P = 0.037. E = excitatory; I = inhibitory

*Synaptic analysis corrected for tissue compaction*

The analysis on excitatory and inhibitory synapses was repeated with NeuN^+^ density as covariate to correct for potential effects of tissue compaction. This did not alter the results across all layers as the interaction effect between tissue type and cortical layer remained significant (excitatory: *F*(6,128.67)=2.85, *P*=.012; inhibitory: *F*(6,127.96)=4.47, *P*<.001). This was again driven by layer 6 synaptic losses (excitatory *P*=.017, inhibitory *P*=.007), but no longer by layer 1 (*P*=0.134).

Supplemental references

1. Seewann A, Kooi EJ, Roosendaal SD, Barkhof F, Van Der Valk P, Geurts JJG. Translating pathology in multiple sclerosis: The combination of postmortem imaging, histopathology and clinical findings. *Acta Neurol Scand*. Published online 2009. doi:10.1111/j.1600-0404.2008.01137.x

2. Jonkman LE, Graaf YG de, Bulk M, et al. Normal Aging Brain Collection Amsterdam (NABCA): A comprehensive collection of postmortem high-field imaging, neuropathological and morphometric datasets of non-neurological controls. *NeuroImage Clin*. Published online 2019. doi:10.1016/j.nicl.2019.101698

3. Vogt BA, Nimchinsky EA, Vogt LJ, Hof PR. Human cingulate cortex: surface features, flat maps, and cytoarchitecture. *J Comp Neurol*. 1995;359(3):490-506. doi:10.1002/cne.903590310

4. Schindelin J, Arganda-Carreras I, Frise E, et al. Fiji: An open-source platform for biological-image analysis. *Nat Methods*. Published online 2012. doi:10.1038/nmeth.2019

5. Dzyubenko E, Rozenberg A, Hermann DM, Faissner A. Colocalization of synapse marker proteins evaluated by STED-microscopy reveals patterns of neuronal synapse distribution in vitro. *J Neurosci Methods*. Published online 2016. doi:10.1016/j.jneumeth.2016.09.001

6. Ogawa Y, Yamaguchi I, Kotani K, Jimbo Y. Deriving theoretical phase locking values of a coupled cortico-thalamic neural mass model using center manifold reduction. *J Comput Neurosci*. 2017;42(3):231-243. doi:10.1007/s10827-017-0638-8

7. Sotero RC, Trujillo-Barreto NJ, Iturria-Medina Y, Carbonell F, Jimenez JC. Realistically coupled neural mass models can generate EEG rhythms. *Neural Comput*. 2007;19(2):478-512. doi:10.1162/neco.2007.19.2.478

8. Becker R, Knock S, Ritter P, Jirsa V. Relating Alpha Power and Phase to Population Firing and Hemodynamic Activity Using a Thalamo-cortical Neural Mass Model. *PLoS Comput Biol*. 2015;11(9). doi:10.1371/journal.pcbi.1004352

9. Breakspear M. Dynamic models of large-scale brain activity. *Nat Neurosci*. 2017;20(3):340-352. doi:10.1038/nn.4497

10. Tzourio-Mazoyer N, Landeau B, Papathanassiou D, et al. Automated anatomical labeling of activations in SPM using a macroscopic anatomical parcellation of the MNI MRI single-subject brain. *NeuroImage*. Published online 2002. doi:10.1006/nimg.2001.0978

11. Gong G, He Y, Concha L, et al. Mapping anatomical connectivity patterns of human cerebral cortex using in vivo diffusion tensor imaging tractography. *Cereb Cortex N Y N 1991*. 2009;19:524-536. doi:10.1093/cercor/bhn102

12. Meijer KA, Cercignani M, Muhlert N, et al. Patterns of white matter damage are non-random and associated with cognitive function in secondary progressive multiple sclerosis. *NeuroImage Clin*. 2016;12:123-131. doi:10.1016/j.nicl.2016.06.009

13. Meijer KA, Steenwijk MD, Douw L, Schoonheim MM, Geurts JJG. Long-range connections are more severely damaged and relevant for cognition in multiple sclerosis. *Brain*. 2020;143(1):150-160. doi:10.1093/brain/awz355
